# Lobster and cod benefit from small-scale northern marine protected areas: inference from an empirical before–after control-impact study

**DOI:** 10.1098/rspb.2012.2679

**Published:** 2013-03-07

**Authors:** Even Moland, Esben Moland Olsen, Halvor Knutsen, Pauline Garrigou, Sigurd Heiberg Espeland, Alf Ring Kleiven, Carl André, Jan Atle Knutsen

**Affiliations:** 1 Flødevigen Marine Research Station, Institute of Marine Research, Nye Flødevigvei 20, 4817 His, Norway; 2 Centre for Ecological and Evolutionary Synthesis (CEES), Department of Biology, University of Oslo, PO Box 1066 Blindern, 0316 Oslo, Norway; 3 Department of Natural Sciences, Faculty of Science and Engineering, University of Agder, 4604 Kristiansand, Norway; 4 Association l'Atelier Bleu, CPIE Côte Provençale, 596 Avenue des Calanques, Parc du Mugel, 13600 La Ciotat, France; 5 Department of Biology and Environmental Sciences-Tjärnö, University of Gothenburg, 452 96 Strömstad, Sweden; 6 Department of Environment, County Governor of Aust-Agder, PO Box 788 Stoa, 4809 Arendal, Norway

**Keywords:** before–after control-impact, marine reserves, baseline information, mark–recapture, *Gadus morhua*, *Homarus gammarus*

## Abstract

Marine protected areas (MPAs) are increasingly implemented as tools to conserve and manage fisheries and target species. Because there are opportunity costs to conservation, there is a need for science-based assessment of MPAs. Here, we present one of the northernmost documentations of MPA effects to date, demonstrated by a replicated before–after control-impact (BACI) approach. In 2006, MPAs were implemented along the Norwegian Skagerrak coast offering complete protection to shellfish and partial protection to fish. By 2010, European lobster (*Homarus gammarus*) catch-per-unit-effort (CPUE) had increased by 245 per cent in MPAs, whereas CPUE in control areas had increased by 87 per cent. Mean size of lobsters increased by 13 per cent in MPAs, whereas increase in control areas was negligible. Furthermore, MPA-responses and population development in control areas varied significantly among regions. This illustrates the importance of a replicated BACI design for reaching robust conclusions and management decisions. Partial protection of Atlantic cod (*Gadus morhua*) was followed by an increase in population density and body size compared with control areas. By 2010, MPA cod were on average 5 cm longer than in any of the control areas. MPAs can be useful management tools in rebuilding and conserving portions of depleted lobster populations in northern temperate waters, and even for a mobile temperate fish species such as the Atlantic cod.

## 1. Introduction

Marine protected areas (MPAs) have received increasing attention as tools in fisheries management and conservation. However, examples of replicated experiments that sampled organismal density before and after establishment of MPAs at sites both inside and outside of the MPAs are rare [1–4]. In European waters, the evaluation of MPAs as a realistic fisheries management tool is impeded by the lack of rigorous assessment of protection [5]. A before–after control-impact (BACI) design, with data from replicated MPA and control sites both before and after MPA designation is presently considered the optimal way of assessing effects of protection [1,4].

MPAs are interesting from a scientific perspective, as protection creates opportunities for studying ecological processes and vital rates in absence of harvest mortality. From a management perspective, ecological studies in MPAs are essential in order to monitor their impacts both within and outside their boundaries [6,7]. The implementation of MPAs is commonly perceived as an opportunity cost to certain stakeholder groups, such as commercial and recreational fishers [8,9]. Consequently, scientific evaluation of the degree to which MPAs meet management objectives is important, especially in areas where the use of this management tool is in the early, experimental stages, and where there is minimal prior knowledge regarding expected outcomes.

Species that are expected to demonstrate the strongest response to protection are those that are subject to high fishing mortality and have low rates of movement relative to the size of the refuge [10–12]. Laurel & Bradbury [13] noted that most research on the design, implementation and evaluation of MPAs is based on small-scale tropical examples. Based on their review of pelagic larval duration and genetic homogeneity in a number of temperate fish species, they proposed that MPAs in high latitudes and cold ocean regions will need to be larger in order to scale with dispersal and gene flow. This assumption has been increasingly challenged by observations of genetically and demographically structured populations of northern species with considerable potential both for long-range dispersal and egg/larval drift (e.g. *Clupea harengus* [14]; *Gadus morhua* [15], *Gadus macrocephalus* [16]). For coastal Atlantic cod (studied herein), it has been shown that fine-scale population structure is maintained by retention of eggs and larvae in fjords and also limited movement of older fish [17,18]. While MPAs with potential to protect temperate species throughout their life histories will need to be large in more open systems, it is not known whether small-scale MPAs may confer benefits to demersal species with pelagic larval stages along convoluted coastlines in high latitudes.

To date, there is a paucity of information regarding responses to protection for Atlantic cod within MPAs throughout the species range. One of the few MPAs under study since 2005 is in Gilbert Bay, Canada [19]. Here, the resident cod stock has been subject to even further decline after the designation of the MPA [20]. Jaworski *et al.* [21] showed that an Icelandic offshore trawl and long-line exclusion zone had a positive effect on abundance of exploitable sizes of Atlantic cod.

Here, we apply a BACI study design to assess the effect of MPAs on lobster and cod in a northern temperate marine ecosystem. As far as we are aware, only few studies have previously used this recommended design and none in northern temperate coastal regions. We show that both lobster and cod generally responded positively to protection, but also that there were clear regional differences in MPA-response and population development in adjacent fished areas. These differences illustrate the value of the BACI study design for science and management.

## 2. Material and methods

### (a) European lobster

The European lobster is a large long-lived decapod crustacean of ecological and commercial importance, distributed from the north of Norway to Morocco in North Africa [22]. European lobster longevity may potentially span several decades [23]. Based on data from wild caught females, size at 50 per cent maturity (i.e. when 25% of females are ovigerous to allow for biennial spawning) in Skagerrak is 79–80 mm carapace length (CL) (≈23 cm total length, TL) (M. Ulmestrand, Swedish University for Agricultural Sciences 2008, unpublished data). In Norway, landings of European lobster (*Homarus gammarus*) decreased dramatically (90%) between 1960 and 1980, indicating a collapse [24]. However, catch rate (catch-per-unit-effort, CPUE), which has decreased by 65 per cent from the 1950s to 2000s [25], is considered a better indication of stock status [26]. Since 2006, this species has ranked on the Norwegian red list as ‘near threatened’ according to the International Union for Conservation of Nature (IUCN) criteria [27]. In Norway, lobsters are legally caught when greater than or equal to 25 cm TL (minimum legal size, MLS) in traps fitted with two circular escape vents measuring 60 mm in diameter. There is a trade and landings ban on egg-bearing females. Effort is limited to 10 and 100 traps for recreational and commercial participants, respectively.

### (b) Atlantic cod

The Atlantic cod is an important food fish for humans, with a wide North Atlantic distribution. Atlantic cod exhibit a range of movement behaviours, from long-distance spawning migration of oceanic life-history forms to stationary coastal cod [18]. Spawning along the coast usually takes place from January to April, depending on temperature [28]. In Skagerrak, coastal cod is genetically structured into local populations on a scale of 30 km or less [15]. Among these populations, age at 50 per cent maturity varies from 2 to 4 years; whereas body length (BL) at 50 per cent maturity varies from 35–60 cm [29]. In Norway, coastal cod is legally caught when greater than or equal to 40 cm (MLS) by the full range of gear, the most common being hook and line, gillnet, fyke net and traps. Coastal cod are also harvested as by-catch by coastal shrimp trawlers. In Skagerrak, several coastal areas have been depleted of adult cod over the last decade [30,31], and a recent study found that 50 per cent of potentially mature cod were removed by fishing each year [32], suggesting a high level of fishing pressure. Since 2002, recruitment of gadoids has been exceptionally poor along the Skagerrak coast, possibly linked to concurrent changes in the plankton community [33].

### (c) Study system

Located on the Norwegian Skagerrak coast, the MPAs studied herein were established to generate knowledge on the development of lobster populations in areas unaffected by extractive fishing. Capture of lobster has been effectively banned in the MPAs since September 2006 through gear restrictions, with only hook and line fishing allowed in the protected areas [25]. Policing of the MPAs are based on collaboration between the Directorate of Fisheries, the Coast Guard and local police.

**Figure 1.**
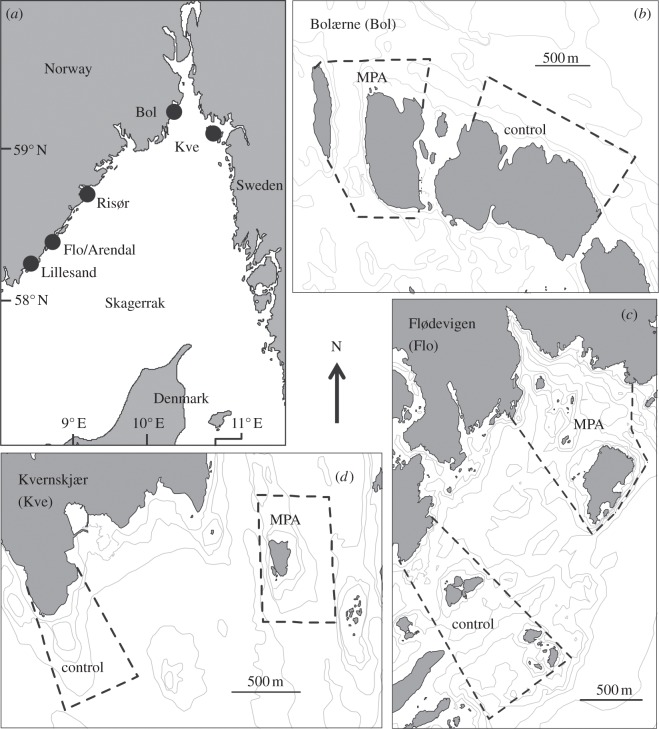
Clockwise from top: (*a*) the location of MPA and control area pairs (Flo, Bol, Kve) and cod sampling sites (Lillesand, Arendal, Risør) in Skagerrak, (*b*) the Bolærne MPA and control area, (*c*) the Flødevigen MPA and control area, and (*d*) the Kvernskjær MPA and control area. For detailed geographical information on cod sampling sites see the electronic supplementary material, figures S1–S3.

The three sites studied, listed from west to east in Skagerrak, are the MPAs located in: (i) Flødevigen (58°25′ N, 8°45′ E), (ii) the Bolærne archipelago at the mouth of the Oslo fjord (59°13′ N, 10°31′ E), and (iii) the small island Kvernskjær (59°02′ N, 10°58′ E) in the Hvaler archipelago (figure 1). Control areas are located adjacent to these and separated from MPAs by distances of 1700, 850 and 2250 m (from MPA centre to control area centre) in Flødevigen, Bolærne and Kvernskjær, respectively (figure 1). At each location, MPAs and control areas are of approximately equal size (≈ 1, ≈ 0.7 and ≈ 0.5 km^2^ in Flødevigen, Bolærne and Kvernskjær, respectively). See also §2*e*.

### (d) Lobster sampling design

An annual standardized research trapping survey, including capture–mark–recapture, was conducted inside proposed MPAs during three consecutive years prior to protection. In 2006, in the last sampling season prior to implementation of the MPAs, adjacent control areas were designated and included in the survey (2006–2010). Thus, as of 2006, the assessment programme was designed as a BACI-paired series approach [34]. Lobsters were sampled using standard ‘parlour’ traps deployed in 10–30 m depth throughout the areas sampled. In each year, a set of 25 traps fished for four days in each of the MPA and control areas (*n* = 100 per site and year), with a ≈ 24-h soak time (ST) prior to each trap haul. Sampling effort was somewhat reduced on some occasions owing to severe weather (for details see the electronic supplementary material, table S1). Sampling was conducted between 20 August and 10 September in each year, during the same week in each region in each year, and simultaneously inside MPAs and control areas in each year (so that shared temporal effects can be accounted for) since inclusion of control areas (2006).

Lobsters were measured and tagged immediately upon capture and released at the site of capture. TL (mm) was measured from the tip of the rostrum to the posterior margin of the telson. All lobsters caught were tagged with individually numbered T-bar anchor tags (TBA2, 45 × 2 mm, Hallprint Pty. Ltd, Holden Hill, South Australia) with printed information about the ongoing project. Tags were inserted in the ventral musculature between cephalotorax and abdomen, to the right side of the midline using a standard tag applicator.

### (e) Cod sampling design

An independent study on cod was conducted on the Norwegian Skagerrak coast during 2005– 2010 [18,35]. Cod were captured in fyke nets (traps) in shallow water (at a depth of 1–10 m) during April to July (see the electronic supplementary material, figures S1–S3). Sampling effort ranged from 74 to 411 trap hauls among years and sites (mean = 228 hauls; for details see the electronic supplementary material, table S2). Trap ST was typically up to one week (range 1– 25 days). Individual cod was measured to the nearest cm, tagged, and released at the site of capture [35]. The cod data includes the Flødevigen MPA, a nearby control area (Arendal), a distant control area 30 km to the southwest (Lillesand) and a distant control area 40 km to the northeast (Risør) (figure 1), during 2 years prior to protection (2005–2006) and 4 years after protection (2007–2010).

### (f) Lobster data analyses

Data analyses and plotting of results were conducted using the R software (v. 2.14.2; [36]). Our hypothesis was that lobster would benefit from the MPA designations owing to the protection arising from prohibition of fixed fishing gears (i.e. any gear with potential to capture this species). This hypothesis was explored by analysing spatial and temporal variation in lobster CPUE and size. We excluded the years 2004 and 2005, from which we had baseline data from MPAs only, in these analyses to obtain a balanced BACI design.

As the CPUE data were skewed towards counts of zero lobsters per trap per day (see the electronic supplementary material, figure S4*a*), we used a zero-inflated Poisson regression model (function: zeroinfl in R, [37]) to analyse the effects of year, treatment and region on CPUE. Year, treatment and region were modelled as factors, where year included five levels (2006–2010, with 2006 (before MPA designation) as the reference level), treatment included two levels: (i) MPA, and (ii) control area, and region included three levels: the MPA and control area pairs (i) Flødevigen, (ii) Bolærne, and (iii) Kvernskjær. Our primary aim was to test for a significant interaction effect between year and treatment, and to test region as the predictor of excess zeros. A significant interaction effect would imply that population changes in time varied between MPAs and control areas. The rationale for testing region as the predictor of excess zeros was the fact that the Flødevigen replicate (both MPA and control area) retained a high proportion of empty traps (zero CPUE counts) throughout the dataset. We compared this model with a generalized linear Poisson regression model predicting CPUE from year × treatment. The Vuong test [38] was used to test whether the zero-inflated model was a significant improvement over a standard Poisson model.

We analysed the body size data by using analysis of variance (ANOVA), with the same factors and factor levels as for the CPUE data (above). Prior to ANOVA, we tested for heterogeneity of variance using Cochran's test [39] (see the electronic supplementary material, table S3). Heterogeneity of variance among treatments may be expected in this type of experiment as variance will increase with the mean. Thus, an increase in mean body size over time, as we expected in MPAs, will result in increased overall variance in the MPA treatment relative to the control area treatment. However, with large sample size and balanced sampling design, heterogeneity of variances should not pose problems [39]. The body size data conformed to the normality assumption of ANOVA (see the electronic supplementary material, figure S4*b*).

### (g) Cod data analyses

Our hypothesis that cod would benefit from the MPA owing to the partial protection arising from restrictions on fishing gear was explored by analysing spatial and temporal variation in cod catch (CC) and cod size using generalized linear models [40]. With reduced fishing, we expected both population density and mean size of individuals to increase over time. CC served as a proxy for population density. There were a substantial number of empty hauls (24%) and most hauls with a catch contained only a few cod, causing CC as a variable to be highly skewed and zero-inflated. Therefore, CC was analysed as a binary process (i.e. the probability of catching at least one cod in a specific haul):2.1
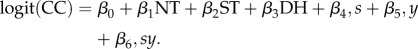


Our primary aim with this model structure was to test for a significant interaction effect (*β*_6_) between site (*s*) and year (*y*), as a significant interaction effect would imply that population changes in time varied between the MPA and control sites. Both site and year were modelled as factors, where site included four levels: (i) Flødevigen MPA, (ii) Arendal control area, (iii) Lillesand control area, and (iv) Risør control area. Furthermore, we aimed to statistically control for the number of traps connected in one haul (NT), the ST and the day of year the trap was hauled (DH). The number of traps connected (in a string) ranged from 1 to 6 (mean 1.7), whereas ST ranged from 1 to 25 days (mean 5.3). Sampling season was 1 April to 20 July (mean 31 May). The NT and ST variables were log-transformed in order to stabilize the variance. Note that the first year of sampling (2005) was not included in this analysis of CC, because the number of traps was not consistently registered, only the number and size of fish. We compared this starting model with simplified alternative models excluding the year effect and/or the site effect, using Akaike information criterion (AIC) as model selection criterion [41]. Next, we tested for effects of site (*s*) and year (*y*) on the mean size (BL) of cod, using the following model structure:2.2
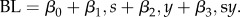


Here, all years (2005–2010) were included as factor levels. In addition to mean size, we also estimated the large-size component of the CC as the 90 per cent percentile length (the length that 90% of the fish are smaller than).

## 3. Results

### (a) Lobster population- and life-history changes

**Figure 2.**
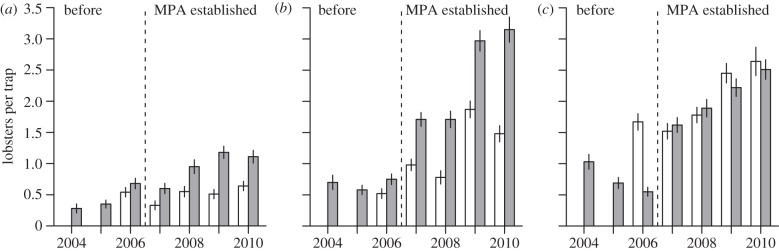
Mean annual number of lobsters per trap per day (CPUE) in proposed and designated MPAs (grey) and control areas (white) (*a*) in Flødevigen, (*b*) Bolærne and (*c*) Kvernskjær sites. Dashed line, MPAs established. Error bars are ±1 standard error (s.e.).

During 2006–2010, a total of 2074 and 1681 lobsters were captured (including recaptures), measured and tagged in the MPAs and control areas, respectively (see the electronic supplementary material, table S1). There was temporal change in CPUE, which varied significantly between MPAs and controls, and also among the three study regions (figure 2*a–c*).

The zero-inflated Poisson regression model predicting CPUE from year, treatment and region was statistically significant (*χ*_11_^2^ = 730.26, *p* < 0.0001). Importantly, the year × treatment interaction effect was significant meaning that the effect of year depended on treatment and that population development was different between treatments (see the electronic supplementary material, table S4). The predictor of excess zeros, region, was statistically significant and, as expected, Flødevigen was significantly different from the other two replicates with regard to excess zeros (*p* < 0.05). The Vuong test suggested that the zero-inflated model was a significant improvement over a standard Poisson regression model (*p* < 0.0001). In the Flødevigen MPA, a modest increase in CPUE was evident in year 2 of protection (2008), and onwards whereas the rate of change was negligible in the control area (figure 2*a*). CPUE was similar in the Bolærne MPA and control area before MPA designation (2006) with increasing difference in all years after MPA designation (2007–2010, figure 2*b*). At Kvernskjær, CPUE was considerably higher in the control area before MPA designation. However, CPUE in the MPA increased rapidly with a mean that was more or less equal to the control area in 2007. During 2008–2010 mean CPUE increased at a similar rate in both areas (figure 2*c*).

**Figure 3.**
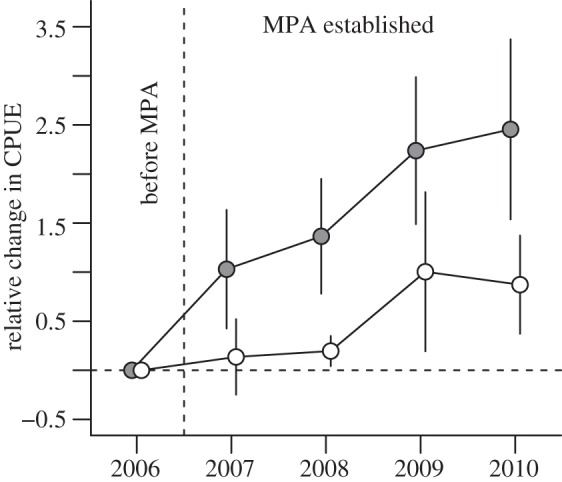
Mean relative change in lobsters per trap per day (catch-per-unit-effort, CPUE) in MPAs (grey) and control areas (white) after MPA designation. Relative change in CPUE was calculated as a ratio of the observed (*t* = *x* (2007–2010)) versus initial values in the year before designation (*t* = 0 (2006)). Average relative change for the three regions was expressed as mean relative change in CPUE ± 1 standard error (s.e.). Vertical dashed line, MPAs established. Error bars are ±1 standard error (s.e.).

For an overall evaluation of temporal change in lobster CPUE in MPA treatments versus controls, we pooled the three study regions. By 2010, the mean relative CPUE had increased by 245 per cent in MPAs, whereas mean relative CPUE in control areas had increased by 87 per cent (figure 3).

**Figure 4.**
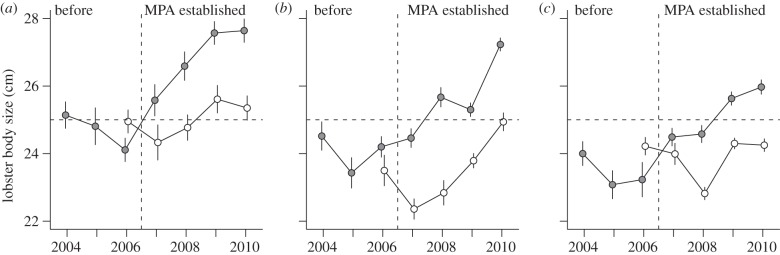
Mean annual body size of lobsters (TL) in proposed and designated MPAs (grey) and control areas (white) in (*a*) Flødevigen, (*b*) Bolærne and (*c*) Kvernskjær. Vertical dashed line, MPAs established; horizontal dashed line, minimum legal size, 25 cm (TL), introduced in 2008 (increased from 24 cm). Error bars are ±1 standard error (s.e.).

Mean body size of lobsters varied significantly among years, depending on treatment (i.e. a significant two-way interaction effect, electronic supplementary material, table S3). From 2006 (before MPA designation) to 2010 (year four of protection), mean body size (TL) of lobsters sampled increased by 14.6 per cent, 12.5 per cent and 11.8 per cent in the Flødevigen, Bolærne and Kvernskjær MPAs, respectively (figure 4). Increase in control areas over the same period was 1.6 per cent, 6.1 per cent and 0.1 per cent at Flødevigen, Bolærne and Kvernskjær, respectively (figure 4). For the three study regions combined, mean body size increased by 13.0 per cent and 2.6 per cent in MPAs and control areas, respectively.

During the four years after MPA designation (2006–2010), a total of 85 lobsters tagged inside MPAs were subsequently reported by recreational and commercial fishers outside MPAs. Fifty-one of the reported recoveries included information on geographical capture position. Out of these, 42 (80%) recoveries were made less than 5 km from MPA centres, although individuals were recovered up to 22 km away (see the electronic supplementary material, figure S5). During the period, four lobsters (4.7% of all reported recoveries) tagged inside MPAs were reported captured in adjacent control areas. This occurred in the Kvernskjær (*n* = 2) and Bolærne (*n* = 2) control areas. No lobsters tagged in the Flødevigen MPA were reported recaptured in the adjacent control area. No lobsters tagged in control areas were recaptured in adjacent MPAs during research trapping surveys.

### (b) Cod population- and life-history changes

**Figure 5.**
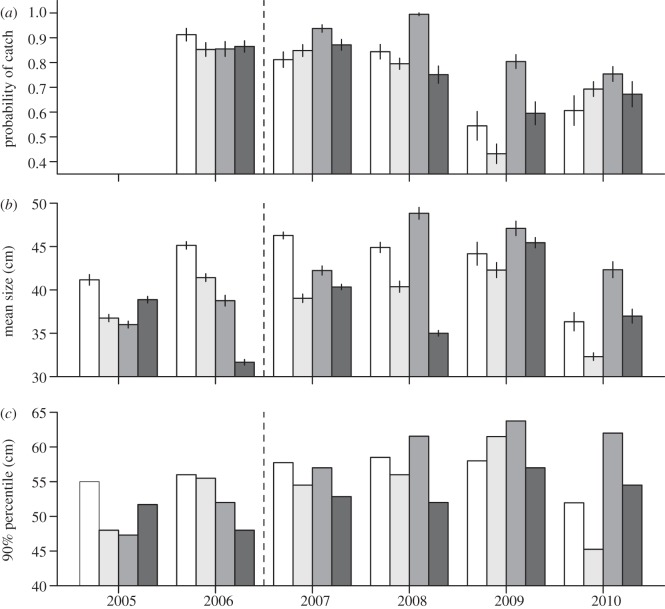
Cod catches (CCs) within the Flødevigen MPA (grey), a nearby control area (light grey), a distant southwestern control area (white) and a distant northeastern control area (dark grey). (*a*) Estimates refer to the probability of a trap containing at least one cod, (*b*) for a mean soak time of 5 days and the mean sampling date of 31 May as predicted from a generalized linear model (see §2), the mean body length of cod and (*c*) the large-size (90% percentile) component of the CC. Dashed line, MPA established. Error bars are ±standard error (s.e.). Map of sampling regions: figure 1; maps of exact sampling locations: electronic supplementary material, figures S1–S3.

During 2005–2010 a total of 12 116 cod were captured and measured (see the electronic supplementary material, table S2). Model selection supported an interaction effect between year and site on the probability of catching cod in a haul (see the electronic supplementary material, table S5). Removing the interaction term or any of the additive effects increased the AIC value by at least 55 units and was, therefore, not supported. In 2006, prior to protection, the expected proportion of traps containing cod was high with no clear differences among sites (figure 5*a*). After designation, during 2007–2010, the MPA consistently had the highest expected proportion of traps with cod (figure 5*a*). In general, the catches tended to decrease throughout the study duration, but the MPA maintained the highest level of traps with cod (figure 5*a*). As expected, the probability of catching cod in a haul also increased with the number of traps used per haul (*β*_2_ = 0.57, s.e. = 0.17, *p* < 0.001) and ST (*β*_3_ = 0.74, s.e. = 0.065, *p* < 0.001). The probability of catching cod also decreased as the sampling season progressed (*β*_4_ = –0.035, s.e. = 0.0029, *p* < 0.001). The mean BL of cod varied significantly among years and sites (*p* < 0.001, electronic supplementary material, table S6). Prior to designation, cod from the MPA were, on average, among the smallest in the study. From 2008 and onwards, the MPA cod had the highest average size (figure 5*b*). In 2010, cod in the MPA were on average 5 cm longer than cod in any of the control regions (figure 5*b*). Similarly, prior to protection, the large-size component (90% percentile length) of cod in the MPA was among the smallest in the study. From 2008 and onwards, the MPA had the largest 90 per cent percentile length (figure 5*c*). In 2010, the large-size component in the MPA was 8 cm longer than in any of the control regions, and 17 cm longer than cod from the nearest control region surrounding the reserve (figure 5*c*).

## 4. Discussion

This study is one of few to use the recommended BACI-approach to assess MPA effects [4], and, to the best of our knowledge, the first to do so for European lobster and Atlantic cod. This approach allowed an unambiguous test of effects of protection on population density and body size of lobster and a relatively robust test of the same for cod. Importantly, our study revealed differences in the response to protection among MPA replicates. Inherently, such differences would not have been detected by a study focusing on a single MPA–control area pair.

Based on our BACI-approach, the findings reported herein provide evidence that protection resulted in significant population change, in the case of lobster, within all three MPAs studied. The average magnitude of change based on CPUE (245%) seems to match reasonably well with that reported in a recent study that addressed effects of protection on European lobster in the southern UK, near the middle of the species' range [42]. That work suggested a 127 per cent increase in the abundance of legal-sized lobsters in the Lundy Island no-take zone, resulting in legal-sized lobsters being five times more abundant within the no-take zone, compared with near and far control areas, after 4 years of protection. Together, these results suggest that MPAs can be an effective management tool in rebuilding and conserving depleted European lobster populations throughout this species' range.

The general pattern of increasing abundance and size of lobsters within the three MPAs is consistent with increased survival of resident individuals owing to cessation of harvesting. A recent acoustic tagging study conducted within the Flødevigen MPA suggested high site fidelity of adult lobsters in this area, with 95 per cent of tagged lobsters remaining within or near MPA boundaries for up to 1 year [43]. This is corroborated by the accumulation of tagged individuals in catches over time (unpublished data, to be reported elsewhere). Although sparse, the reports of recoveries outside MPAs (=85 lobsters, constituting ≈ 4% of the tagged population in MPAs, see the electronic supplementary material, figure S5), are indicative of some degree of spill-over to adjoining areas and existence of more transient behaviours. The mean increase in body size in the three MPAs was 13.0 per cent, not an uncommon response in MPAs elsewhere (see Lester *et al*. [44] and references therein), and indicative of an ongoing recovery of the age and size structure which is likely to benefit the reproductive potential within the MPAs. A modest increase in mean body size (2.6%) was also evident in the control areas. This increase could be owing to the introduction of a 25 cm (TL) MLS (increased from 24 cm) in 2008. In general, lobster species have shown rapid positive response to protection within MPAs [42,45,46].

Coastal Atlantic cod responded significantly to partial protection (absence of harvesting by traps and gillnets) through increased population density and body size. Coastal cod are genetically structured on a fine geographical scale [15,18], implying that populations are also demographically disconnected at a fine scale. Thus, reduction of fishing mortality on a small spatial scale can be sufficient to drive population and life-history change in this species. There are no studies on Atlantic cod to which we can compare our results. However, evidence for effects of small-scale MPAs on density and body size of mobile predatory fish species is abundant from other temperate areas (see Lester *et al*. [44] and references therein).

Recent studies on Skagerrak coastal cod have demonstrated that harvest selection may act on life-history traits (such as growth) as well as on behavioural traits [32,47]. The results presented herein provide support to the notion that MPAs may help to counter evolutionary impacts of harvesting on cod through restoration of size–structure [48]. However, MPAs may also set up new and unanticipated selection pressures, if, for example, survival favours individuals with limited space use. The positive effect of partial protection on cod in the small Flødevigen MPA could be attributed to increased survival in individuals displaying extreme site fidelity, a behaviour that might not be representative for the population norm. This should be considered when designing MPAs, as they should ideally be configured to accommodate a range of space use behaviours.

Although lobster population density increased in all MPAs, our study revealed differences in response to protection among MPA/control area replicates (see figure 2 and §3). The easternmost replicate (Kvernskjær) stood out with a population density that was higher in the control area before MPA designation. Here, population density in the MPA increased rapidly and was more or less equal to that of the control area in 2007 (1 year after MPA designation). Thereafter, population density increased at a similar rate in both areas (figure 2*c*). Similarly, albeit of lesser magnitude, population density increased in the Bolærne control area. Here, the increased population density observed in the 2009 sampling season might have been caused by the introduction of an increased MLS in 2008. This seemed to have constituted a temporary conservation effect that was removed by the time we conducted the 2010 sampling (figure 2*b*). The adjacent Bolærne MPA was the replicate in which we observed the largest effect on lobster population density throughout the study. Effects were present, but more modest in the westernmost and largest MPA (Flødevigen). These differences demonstrate spatial heterogeneity in effects of protection in largely similar systems that cannot be given a straightforward explanation. We deem this an important finding in itself when considering that scientific studies are used to support management decisions that may have significant socioeconomic impacts. This study thus underscores the need for proper design choices and adequate replication in studies assessing MPA effects [1–4].

Importantly, MPAs should not be viewed as a cure all solution to conserve depleted fish stocks, as there might be other large-scale effects related to global change and subsequent community changes in coastal seas, acting in addition to harvesting and proximately causing the observed decline and recruitment failure [33,49]. Because of this, cod populations might be rendered less robust to harvesting than they were historically. That said, properly designed experimental evaluation of MPAs (including no-take areas) at spatial scales that are relevant to fisheries management are urgently needed to obtain good knowledge on the full potential of this management tool in northern waters.

Coastal areas face management challenges for lobster and cod. For lobster, recreational fishing is popular, regulations are liberal and reported landings do not always reflect the actual harvest [26]. In conclusion, our study show that harvested marine species in northern temperate waters may benefit from small-scale MPAs. We further emphasize the need for replicated MPA/control area pairs (i.e. the BACI-paired series design) in the assessment of MPAs, as monitoring single MPAs may lead to variable conclusions and, perhaps, management implications.
